# Characterization of a Novel Anti-Human HB-EGF Monoclonal Antibody Applicable for Paraffin-Embedded Tissues and Diagnosis of HB-EGF-Related Cancers

**DOI:** 10.1089/mab.2015.0062

**Published:** 2016-04-01

**Authors:** Ryo Iwamoto, Mika Takagi, Jun-ichi Akatsuka, Ken-ichiro Ono, Yoshiro Kishi, Eisuke Mekada

**Affiliations:** ^1^ Department of Cell Biology, Research Institute for Microbial Diseases, Osaka University, Osaka, Japan.; ^2^ Medical & Biological Laboratories Co., Ltd, Nagoya, Japan.

## Abstract

Heparin-binding EGF-like growth factor (HB-EGF) is a member of the EGF family of growth factors that bind to and activate the EGF receptor (EGFR/ErbB1) and ErbB4. HB-EGF plays pivotal roles in pathophysiological processes, including cancer. Thus, monoclonal antibodies (mAbs) for HB-EGF detection could be an important tool in the therapeutic diagnosis of HB-EGF-related cancers and other diseases. However, few mAbs, especially those applicable for immunohistochemistry (IHC), have been established to date. In this study, we generated a clone of hybridoma-derived mAb 2-108 by immunizing mice with recombinant human HB-EGF protein expressed by human cells. The mAb 2-108 specifically bound to human HB-EGF but not to mouse HB-EGF and was successful in immunoblotting, even under reducing conditions, immunoprecipitation, and immunofluorescence for unfixed as well as paraformaldehyde-fixed cells. Notably, this mAb was effective in IHC of paraffin-embedded tumor specimens. Epitope mapping analysis showed that mAb 2-108 recognized the N-terminal prodomain in HB-EGF. These results indicate that this new anti-HB-EGF mAb 2-108 would be useful in the diagnosis of HB-EGF-related cancers and would be a strong tool in both basic and clinical research on HB-EGF.

## Introduction

Heparin-binding EGF-like growth factor (HB-EGF) is a member of the EGF family of growth factors that bind to and activate the EGF receptor and ErbB4.^(1,2)^ Like other members of the EGF family, HB-EGF is synthesized as a membrane-anchored protein (proHB-EGF), which is composed of a signal peptide, a propeptide, and heparin-binding, EGF-like, juxtamembrane, transmembrane, and cytoplasmic domains.^(3)^ proHB-EGF is biologically active as a juxtacrine growth factor that signals to neighboring cells in a nondiffusible manner^(4,5)^ and also functions as the receptor for the diphtheria toxin (DT).^(6,7)^ proHB-EGF is cleaved at its juxtamembrane domain by metalloproteases in a process called ectodomain shedding.^(8)^ Ectodomain shedding of proHB-EGF yields a soluble form of HB-EGF (sHB-EGF), which is a potent mitogen and chemoattractant for cells expressing the cognate ErbB receptor.^(9,10)^

Several lines of evidence have implicated HB-EGF in cancer cell proliferation, malignancy, metastatic potential, and chemotherapy resistance.^(11–17)^ HB-EGF expression is elevated in many types of malignant tumors.^(12,18–21)^ In ovarian cancer, HB-EGF expression was increased in advanced cancer compared with normal ovary tissue^(12)^ and associated with poor clinical outcome.^(13)^ HB-EGF is not only expressed in cancer cells but also in cancer-surrounding stroma to involve tumor progression.^(22)^ Thus, HB-EGF is recognized as a possible target for cancer therapy, and anti-HB-EGF antibody^(23)^ and CRM197 (a nontoxic mutant form of DT that neutralizes HB-EGF activity)^(24,25)^ are undergoing clinical development as anticancer drugs.^(26)^

Monoclonal antibodies (mAbs) available for HB-EGF detection could be an important tool in the diagnosis of HB-EGF-related cancers and other diseases. Although a number of mAbs reacting to HB-EGF have been isolated,^(23,27)^ those especially applicable in immunohistochemistry (IHC) of paraffin-embedded specimens have not been established. In this study, we generated mAbs to HB-EGF and obtained a clone of hybridoma that detects HB-EGF both in intact cells and fixed paraffin-embedded sections. In this study, we characterize this antibody and demonstrate its usefulness for several applications. Our results suggest that this new anti-HB-EGF mAb 2-108 would be a powerful tool in the therapeutic diagnosis of HB-EGF-related cancers and other diseases.

## Materials and Methods

### Materials

The mouse anti-human HB-EGF mAb 4G10 was prepared as described previously.^(27)^ The goat anti-mouse HB-EGF polyclonal antibody (pAb) M-18 was obtained from Santa Cruz Biotechnology, Inc. (Dallas, TX). CRM197 was prepared as described previously.^(28)^

### Preparation of anti-HB-EGF mAbs

An extracellular domain (amino acids 1–161) of human HB-EGF protein was expressed in HEK293T cells and purified from the culture supernatant. BALB/c mice (4–6 weeks old) were immunized with the purified recombinant HB-EGF extracellular domain. After six immunizations, spleen lymphocytes were collected and fused with P3U1 myeloma cells in a 50% polyethylene glycol 4000 solution (Wako, Osaka, Japan). The fused cells were plated in 96-well plates in the RPMI-1640 medium containing 15% fetal calf serum (FCS; Equitech-Bio, Inc., Kerrville, TX), penicillin/streptomycin (Invitrogen, Thermo Fisher Scientific, Waltham, MA), and HAT solution (Invitrogen). After 10 days of incubation at 37°C with 5% CO_2_ in a humidified environment, culture supernatants were collected and screened for their ability to bind to the recombinant human HB-EGF immobilized on 96-well plates using indirect enzyme-linked immunosorbent assay (ELISA). Selected positive hybridoma colonies were expanded and subcloned by limiting dilution. Purification of the anti-human HB-EGF antibody (clone: 2-108; Medical & Biological Laboratories [MBL] Co., Ltd, Nagoya, Japan) was performed with protein A affinity chromatography (GE Healthcare, Buckinghamshire, UK). The immunoglobulin subclass of 2-108 (IgG1) was determined with anti-mouse isotype-specific antibodies (MBL). Clone 2E12 was purchased from MBL as a mouse IgG isotype control (M075-3).

### Cell culture and transfection

Monkey Vero, Vero-H (Vero cells stably expressing human HB-EGF),^(8)^ and Vero-mH (Vero cells stably expressing mouse HB-EGF)^(29)^ cell lines were maintained in Dulbecco's modified Eagle's medium (DMEM) with nonessential amino acids supplemented with heat-inactivated 10% FCS. Human HEK293, SKOV-3, MCAS, and RMG-1 cells were maintained in DMEM with heat-inactivated 10% FCS. Plasmid transfection was performed using the Lipofectamine 2000 Transfection Reagent (Thermo Fisher Scientific) according to the manufacturer's instructions.

### Plasmids

The cDNAs encoding human/mouse HB-EGF chimeric molecules^(25,27)^ were subcloned into a mammalian myc-tagged expression vector, pcDNA6-myc-His (Thermo Fisher Scientific). Point mutations were introduced into pRcHB-EGF,^(6)^ which contains the entire human proHB-EGF coding region, by site-directed mutagenesis using the KOD-Plus-Mutagenesis Kit according to the manufacturer's instructions (Toyobo, Osaka, Japan).

### Immunoblotting

Cells were lysed with a cell lysis buffer (60 mM 1-O-n-octyl-β-D-glucopyranoside, 0.15 M NaCl, 10 mM HEPES-NaOH, pH 7.2, 1/100× protease inhibitor cocktail [Nacalai, Kyoto, Japan]), and centrifuged at 20,000 *g* for 30 minutes. The supernatant was collected, mixed with sodium dodecyl sulfate (SDS) gel sample buffer supplemented with or without 10% β-mercaptoethanol, and loaded onto 15% SDS electrophoresis gels. Separated proteins were electrotransferred to a polyvinylidene difluoride membrane (Immobilon-P; Merck Millipore, Darmstadt, Germany), and the membrane was blocked in Tween 20-Tris Buffered Saline (TTBS) (10 mM Tris-HCl pH 7.5, 0.15 M NaCl, 0.05% Tween20) containing 1% skim milk for 1 hour, followed by incubation with a primary antibody (at 1 μg/mL if not indicated) in TTBS for 1 hour. The membrane was then incubated with a horseradish peroxidase (HRP)-conjugated secondary antibody (1:3000) for 1 hour. Chemiluminescent detection was carried out with the Pierce ECL detection system (Thermo Fisher Scientific) according to the manufacturer's instructions.

### Immunoprecipitation

Cell surface biotinylation and immunoprecipitation of HB-EGF were performed as described previously.^(6)^ In brief, cells were labeled with a biotinylation reagent, sulfo-NHS-biotin (Perbio Science, Erembodegem, Belgium). Surface-biotinylated cells were homogenized in the cell lysis buffer, and the lysate was used for immunoprecipitation with each mAb (4 μg/mL) and Sepharose-conjugated anti-mouse IgG. The precipitated proteins were analyzed by SDS-polyacrylamide gel electrophoresis (PAGE) and western blotting using HRP-conjugated streptavidin (Perbio Science).

### Immunofluorescence

To detect HB-EGF molecules on the surface of intact cells, cells were incubated with 1 μg/mL of each primary mAb at 4°C for 1 hour and then fixed with 4% paraformaldehyde (PFA) in phosphate-buffered saline (PBS) at room temperature for 30 minutes. Cells were then stained with the Alexa546-conjugated anti-mouse antibody (Life Technologies, Thermo Fisher Scientific) at 4°C for 1 hour. Images were captured by a conventional fluorescent microscope (Olympus BX50, Tokyo, Japan). For CRM197 competition, cells were incubated with 50 μg/mL of CRM197 at 4°C for 1 hour before incubation with the primary mAb.

### Cell-binding ELISA

Cell-binding ELISA was performed by the method described previously^(27)^ with a slight modification. Briefly, cells were incubated with each mAb (0.1 μg/mL) in a blocking solution (1% skim milk in PBS containing 0.2 mM CaCl_2_ and 0.2 mM MgCl_2_ (PBS(+)) for 1 hour at 4°C before or after the fixation. Fixation was carried out with 4% PFA in PBS(+). After incubation with HRP-conjugated anti-mouse IgG Ab (Chemicon, Billerica, MA) for 1 hour at 4°C, color development detection was carried out by the POD reagent (Nacalai) according to the manufacturer's instructions. Light emission was measured at 450 nm using a microplate reader (Thermo Electron Corp, Madison, WI). For CRM197 competition assay, fixed or unfixed cells were incubated with 50 μg/mL of CRM197 at 4°C for 1 hour before the incubation with each primary mAb.

### IHC for paraffin-embedded tissues

For the examination of cultured cells, more than 1 × 10^7^ cells of Vero or Vero-H cells were packed by centrifugation (200 *g*), fixed with 4% PFA, and embedded in paraffin. For the examination of mouse tumors, a total volume of 0.1 mL containing 5 × 10^6^ of SKOV-3, MCAS, or RMG-1 cells suspended in serum-free DMEM was subcutaneously injected into nude mice at 5 weeks of age. At 6 weeks after tumor cell injection, the formed tumors were excised, fixed with 4% PFA, dehydrated, and embedded in paraffin. All experimental use of animals complied with the Guidelines for Animal Care of Osaka University. For the examination of human cancer tissues from patients, we purchased the Human Tissue Array (Shanghai Outdo Biotech Co. Ltd., Shanghai, China: ovarian cancer and prostate cancer tissues of human multiple organ tumor, OD-CT-Rp03-002; breast cancer, OD-CT-RpBre03-004; uterus cancer, OD-CT-RpUtr03-002; gastric cancer, OD-CT-DgStm03-003; and colon cancer, HCol-Ade060PG-01) from MBL.

Paraffin-embedded sections (4 μm thick) were deparaffinized and then treated with 10 mM citrate buffer (pH 6.0) at 125°C for 5 minutes by autoclave. Endogenous hydrogen peroxidase was quenched with 3% H_2_O_2_ in PBS at room temperature for 10 minutes. Sections were incubated with 10% goat serum (Nichirei, Tokyo, Japan) at room temperature for 30 minutes, followed by incubation with each mAb at 1 μg/mL in 1% bovine serum albumin (BSA) in PBS overnight at 4°C, and with secondary antibodies Histostar (Ms+Rb) (MBL) for 1 hour at room temperature. To stain the paraffin sections of xenografted ovarian cancer tumors from nude mice, sections were incubated with biotinylated mAb 2-108 (B-2-108) or biotinylated control mAb 2E12 (B-2E12) as a primary Ab at 5 μg/mL in 1% BSA in PBS overnight at 4°C, and with HRP-conjugated streptavidin (Vector, Burlingame, CA) for 30 minutes at room temperature. Staining was developed using a diaminobenzidine substrate (Vector). Sections were counterstained lightly with Mayer's hematoxylin. Images were captured by conventional light microscopy (Nikon Eclipse E400, Tokyo, Japan).

### Epitope mapping

To detect the domains in human HB-EGF recognized by mAb 2-108, HEK293 cells that were transfected with expression vectors encoding human/mouse HB-EGF chimeric molecules were tested by cell-binding assay as described earlier. In these assays, cells were fixed before incubation with the primary mAb. The score of the specific binding of each mAb to HB-EGF chimeric molecules on the cell surface was calculated by subtracting the binding value of mock transfectant from the total binding value. To detect the amino acid residues in human HB-EGF that are recognized by mAb 2-108, HEK293 cells that were transfected with expression vectors encoding human HB-EGF mutants were tested by cell-binding ELISA. The score of the specific binding of mAb 2-108 to HB-EGF mutant molecules on the cell surface was calculated by subtracting the binding value of the mock transfectant from the total binding value, and then the specific binding score was normalized with that of 4G10 mAb, as a standard for the expression of each construct.

## Results

### Production of mouse anti-human HB-EGF mAbs

In this study, we aimed to generate anti-HB-EGF mAbs applicable for IHC analysis. Although the previous study used recombinant protein produced by insect cells as antigen,^(27)^ in this study, recombinant human HB-EGF protein produced by human HEK293 cells was used for the antigen to immunize mice. We obtained a number of clones of hybridomas producing IgGs that recognize the HB-EGF antigen and selected several candidate clones applicable for IHC. Because the selected clones showed essentially similar characteristics in our examinations, here we have characterized mAb 2-108 as a representative among the clones. For the reference antibody, we used mAb 4G10, an anti-HB-EGF mAb that we previously produced^(27)^ and that is now commercially available.

### Immunoblotting

**FIG. 1.**
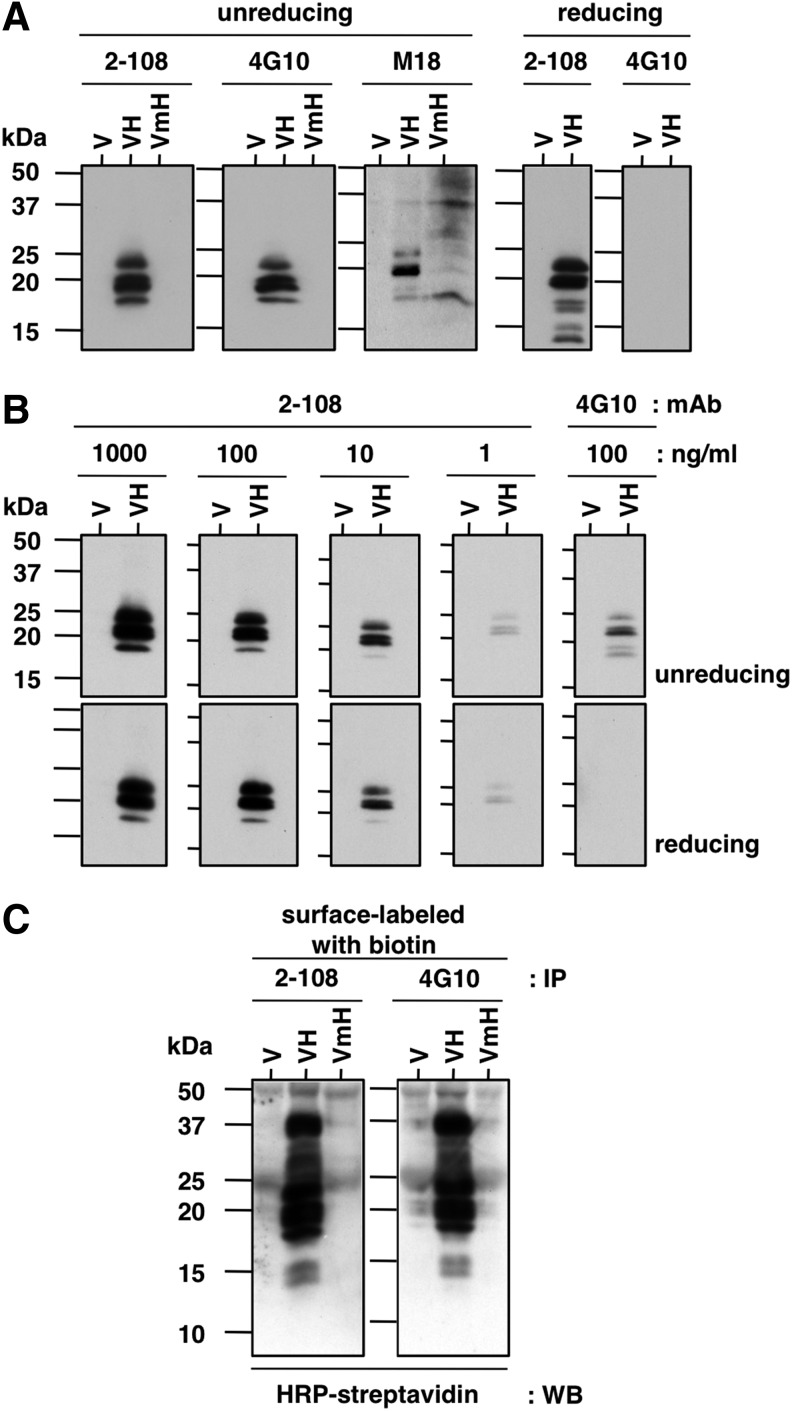
Immunoblotting and immunoprecipitation assays. **(A)** Each mAb (2-108 and 4G10) was tested for immunoblotting against lysates of Vero (V), Vero-H (VH), or Vero-mH (VmH) cells under nonreducing and reducing conditions. Anti-mouse HB-EGF pAb M-18 (M18) was used to confirm mouse HB-EGF expression in Vero-mH cells. In immunoblotting, the differences in band pattern of mouse HB-EGF from that of human HB-EGF are due to differences in post-translational processing and modifications. **(B)** Each mAb (2-108 and 4G10) was tested for immunoblotting against lysates of Vero (V) or Vero-H (VH) cells under nonreducing and reducing conditions at the indicated concentrations. **(C)** Immunoprecipitation assay. Vero (V), Vero-H (VH), and Vero-mH (VmH) cells were labeled by biotin, and cell lysates were immunoprecipitated with each mAb. The precipitated proteins were analyzed by SDS-PAGE and western blotting using HRP-conjugated streptavidin. HB-EGF, heparin-binding EGF-like growth factor; HRP, horseradish peroxidase; pAb, polyclonal antibody; SDS-PAGE, sodium dodecyl sulfate–polyacrylamide gel electrophoresis.

We first examined the applicability of mAb 2-108 for immunoblotting. Cell lysates of Vero, Vero-H, and Vero-mH cells were subjected to SDS-PAGE under nonreducing and reducing conditions and immunoblotting was performed. As shown in Figure 1A, mAb 2-108 could detect human HB-EGF expression in Vero-H cells in both reducing and nonreducing conditions, while 4G10 could only detect human HB-EGF expression in nonreducing conditions, consistent with previous results.^(27)^ M-18, an anti-mouse HB-EGF pAb, detected both human and mouse HB-EGF, but mAb 2-108 was not able to detect mouse HB-EGF, similar to 4G10.^(27)^

Reactivity of mAb 2-108 at 10 ng/mL was slightly stronger than that of mAb 4G10 at 100 ng/mL (Fig. 1B). In our experience, this is the best reactivity in immunoblotting among all HB-EGF mAbs so far.

### Immunoprecipitation

To examine the applicability of this antibody for immunoprecipitation, cell surfaces of Vero, Vero-H, and Vero-mH cells were labeled with the membrane-impermeable biotinylation reagent sulfo-NHS-biotin. Cells were lysed and the lysate was immunoprecipitated with mAb 2-108 or mAb 4G10, followed by SDS-PAGE and immunoblotting with HRP-conjugated streptavidin. As shown in Figure 1C, both mAbs precipitated human HB-EGF but not mouse HB-EGF to a similar degree.

### Detection of intact HB-EGF on the cell surface by immunofluorescence and cell-binding ELISA

**FIG. 2.**
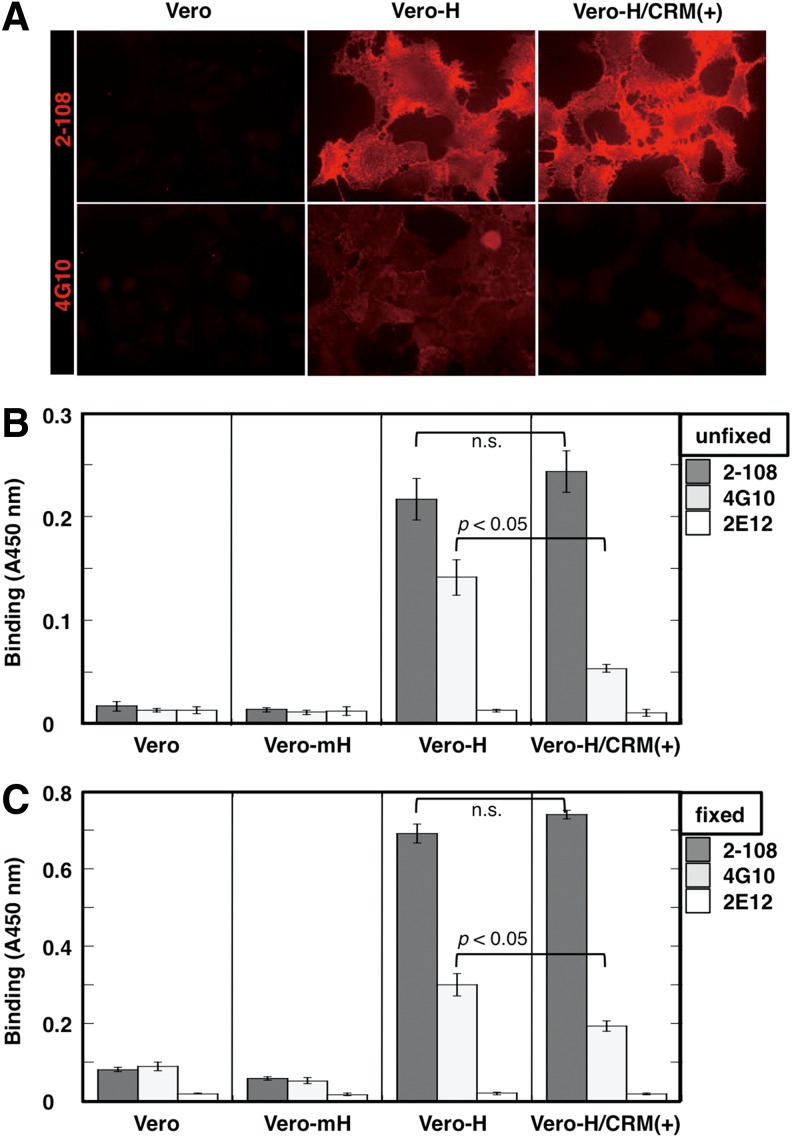
Immunocytochemistry and cell-binding ELISA. **(A)** Each mAb (2-108 and 4G10) was tested for immunocytochemistry against unfixed Vero or Vero-H cells under preincubation with or without CRM197. **(B, C)** Cell-binding ELISA. **(B)** Each mAb (2-108, 4G10, and 2E12 as an isotype-matched control mAb) was tested for cell-binding ELISA against unfixed Vero, Vero-H, or Vero-mH cells under preincubation with or without CRM197. **(C)** Cells were fixed with 4% PFA in PBS before the incubation with each primary mAb. Results are shown in chromogen concentration measured by absorbance of 450 nm. Each bar indicates the mean ± SE (*n* = 3). ELISA, enzyme-linked immunosorbent assay; n.s., not statistically significant; PBS, phosphate-buffered saline; PFA, paraformaldehyde; SE, standard error.

To examine whether mAb 2-108 can bind to intact HB-EGF on the cell surface, we performed immunostaining with mAb 2-108 against unfixed Vero and Vero-H cells. As shown in Figure 2A, mAb 2-108 stained unfixed Vero-H cells more strongly than mAb 4G10, but did not stain the parental Vero cells. In a similar experiment, we confirmed that mAb 2-108 could also stain 4% PFA-fixed Vero-H cells (data not shown). The binding of mAb 2-108 to Vero-H cells was not competitively blocked by CRM197, a nontoxic mutant of DT that specifically binds to the EGF-like domain of HB-EGF,^(25)^ while binding of mAb 4G10 was completely blocked by CRM197 (Fig. 2A). This result suggests that mAb 2-108 recognizes a domain in HB-EGF that is different from the EGF-like domain recognized by both CRM197 and mAb 4G10.^(27)^

To quantitatively measure the above observations, cell-binding ELISA^(27)^ was performed using unfixed Vero, Vero-H, and Vero-mH cells. As shown in Figure 2B, both mAb 2-108 and mAb 4G10 showed specific binding to Vero-H cells, but no binding was observed to Vero and Vero-mH cells. The binding of mAb 2-108 to Vero-H cells was ∼1.5 times higher than that of mAb 4G10 and was not blocked by CRM197, while that of mAb 4G10 was strongly blocked (Fig. 2B). Thus, the cell-binding ELISA quantitatively reproduced the immunofluorescent cell staining results. PFA-fixed cells showed essentially similar results as those in unfixed cells (Fig. 2C). However, the binding competition by CRM197 against mAb 4G10 was weaker than the case of the intact cells, which might be due to the decreased reactivity of CRM197 to HB-EGF by PFA fixation (Fig. 2C). Unexpectedly, overall binding values of mAbs 2-108 and 4G10, but not of 2E10, were higher than those of unfixed cells (Fig. 2C), although the reason remains unclear. Thus, these results indicate that PFA fixation is more suitable to detect HB-EGF staining using this mAb.

### IHC for paraffin-embedded sections

**FIG. 3.**
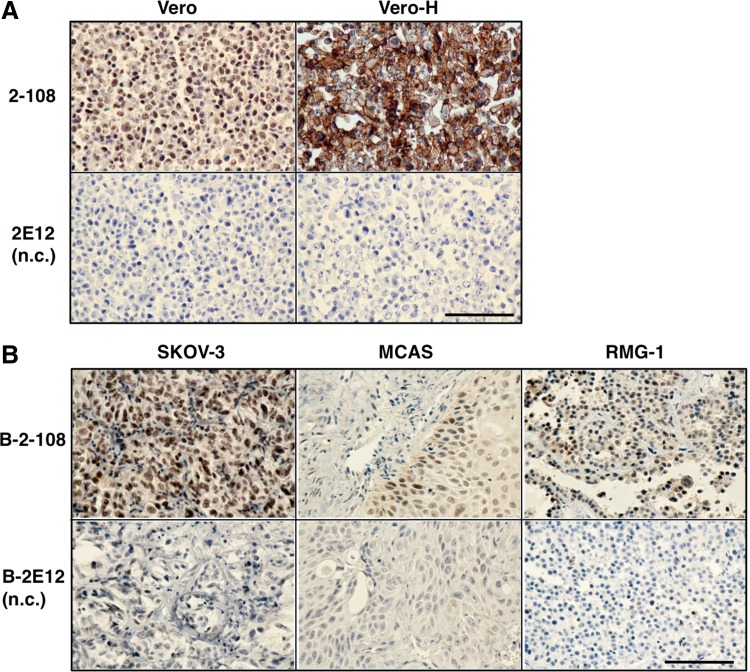
IHC for paraffin-embedded tissues. **(A)** Paraffin-embedded sections of the pellets of Vero or Vero-H cells were stained with mAb 2-108 or mAb 2E12 (n.c., negative control) and counterstained with hematoxylin. **(B)** Paraffin-embedded sections of the xenografted tumors of ovarian cancer SKOV-3, MCAS, or RMG-1 cells in nude mice were stained with biotinylated mAb 2-108 (B-2-108) or biotinylated control mAb 2E12 (B-2E12) and counterstained with hematoxylin. IHC, immunohistochemistry.

To examine whether mAb 2-108 is effective for IHC in paraffin-embedded sections, we first tested staining with mAb 2-108 against paraffin-embedded sections of Vero and Vero-H cell pellets. Compared with the subtype-matched control mAb 2E12, mAb 2-108 strongly stained paraffin-embedded Vero-H cells (Fig. 3A). MAb 2-108 could also weakly stain the parental Vero cells that express monkey HB-EGF at a low level.^(6)^ We next examined the staining of mouse xenografted tumors. We performed IHC by a direct method using biotinylated mAbs to avoid reaction with endogenous mouse IgG in mouse tissues. Biotinylated (B-) mAb 2-108 successfully stained the cancerous regions of xenografted tumors of three ovarian cancer cell lines, SKOV-3, MCAS, and RMG-1, in contrast to the control B-mAb 2E12 (Fig. 3B). While the cancerous regions were clearly stained with B-mAb 2-108, interstitial stroma regions were stained at low levels.

**FIG. 4.**
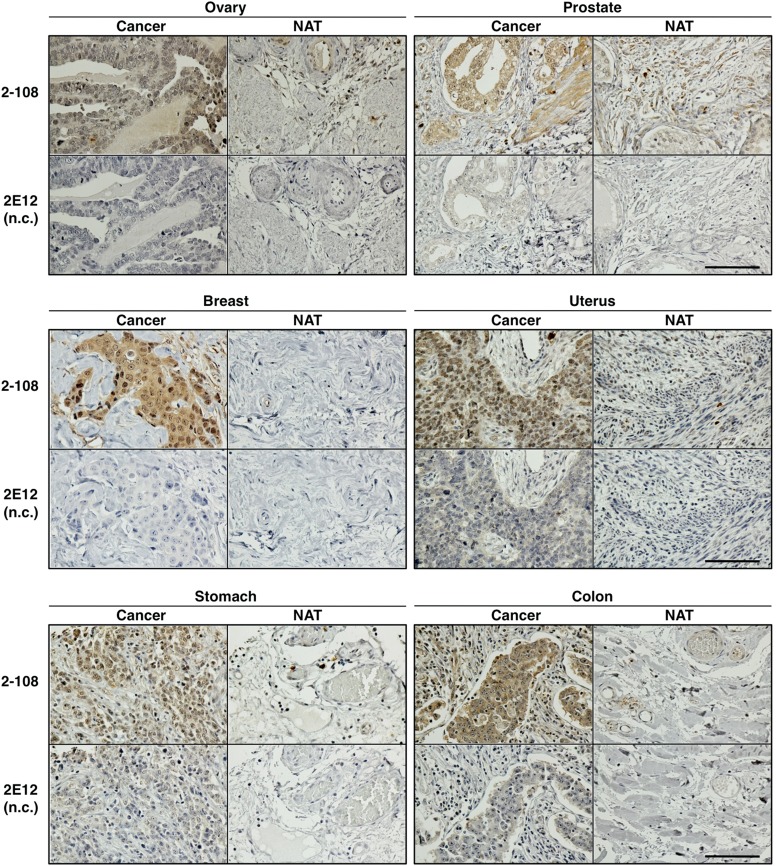
IHC for paraffin-embedded tissues. Paraffin-embedded sections of the tumors (cancer) or the corresponding normal adjacent tissue (NAT) of six cancer patients were stained with mAb 2-108 or control mAb 2E12 and counterstained with hematoxylin. Hematoxylin staining is in blue, and brown grains indicate positive staining for mAb 2-108. Positive signals are observed more abundantly in the cancer cells than the neighboring interstitial stroma areas. Bars: 100 μm for all panels.

Finally, IHC of paraffin-embedded clinical specimens was performed with mAb 2-108 (Fig. 4). Compared with the control mAb 2E12, mAb 2-108 successfully stained paraffin-embedded sections of cancer tumors of ovary, prostate, breast, uterus, stomach, and colon tissues, in which HB-EGF expression has been reported.^(12,20–22,30–32)^ Conversely, each corresponding normal adjacent tissue showed little positive staining with mAb 2-108, suggesting that the positive signals are not due to nonspecific binding of mAb 2-108. These results indicate that mAb 2-108 can recognize HB-EGF in paraffin-embedded sections.

### Epitope mapping

**FIG. 5.**
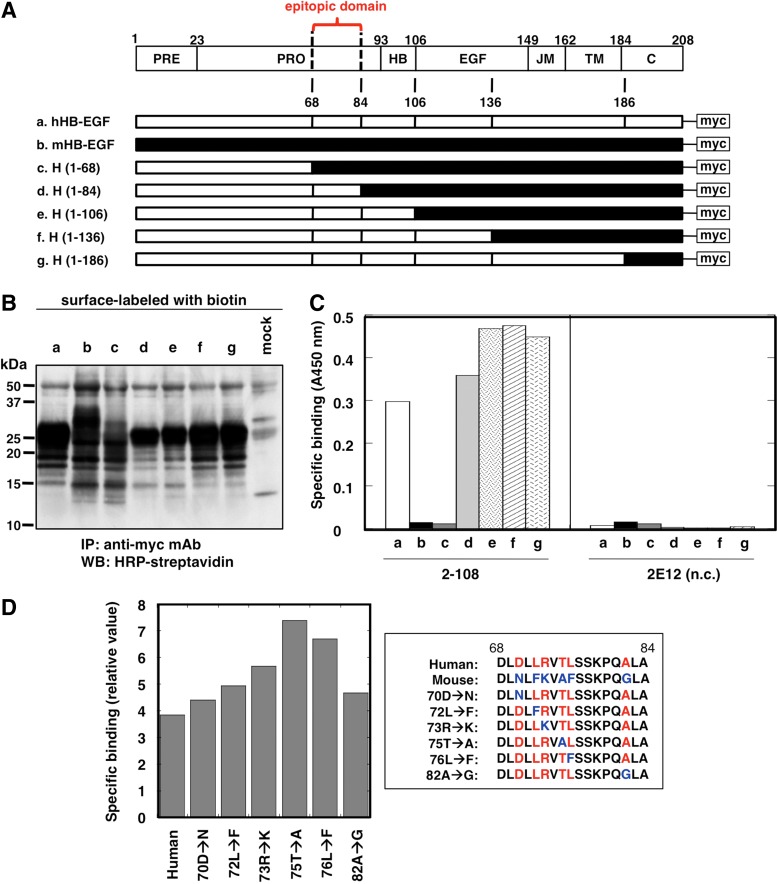
Epitope mapping of mAb 2-108. **(A)** Schematic structures of the human/mouse HB-EGF chimeric molecule constructs. All constructs have a myc-tag in the C-terminus. Epitopic domain revealed in **(C)** is also indicated (red). **(B)** Comparable cell surface expression of each human/mouse HB-EGF chimeric molecule in the transfected HEK293 cells was confirmed by immunoprecipitation assay using anti-myc tag mAb. **(C)** Binding of each mAb (2-108 or 2E12 control mAb) to HEK293 cells expressing human/mouse HB-EGF chimeric molecule constructs as shown in **(A)**. a, human HB-EGF; b, mouse HB-EGF; c, H(1–68); d, H(1–84); e, H(1–106); f, H(1–136); g, H(1–186). Binding of mAbs was measured by cell-binding ELISA, and binding values were determined as described in the Materials and Methods section. Essentially similar results were obtained in three independent experiments. **(D)** Epitope mapping of mAb 2-108 using point mutations between amino acid residues 68 and 84 located in the prodomain of human HB-EGF. (Left) Binding of mAb 2-108 to the HEK293 cells expressing each human HB-EGF mutant construct as shown in the right panel. Essentially similar results were obtained in three independent experiments. (Right) Amino acid sequences in the site between 68 and 84 of human HB-EGF, mouse HB-EGF, and human HB-EGF mutants. mAbs, monoclonal antibodies. PRE, signal sequence; PRO, prodomain; HB, heparin-binding domain; EGF, EGF-like domain; JM, juxtamembrane domain; TM, transmembrane domain; C, cytoplasmic domain.

Our results showed that mAb 2-108 bound to human HB-EGF but not to mouse HB-EGF (Fig. 2B). Taking advantage of species specificity, we determined the epitopic region in human HB-EGF for this mAb using a method similar to a previously described approach.^(27)^ We expressed a series of human/mouse HB-EGF chimeric molecules tagged with the myc epitope (Fig. 5A) in human HEK293 cells. We confirmed that each HB-EGF chimeric molecule was expressed on the surface of transfected cells at comparable levels (Fig. 5B). Binding of mAb 2-108 or control mAb 2E12 to each chimeric HB-EGF molecule was assessed by cell-binding ELISA. As shown in Figure. 5C, 2-108 mAb bound to cells expressing full-length human HB-EGF, H(1–186), H(1–136), H(1–106), and H(1–84), but not to H(1–68)-expressing or mouse HB-EGF-expressing cells. The control mAb 2E12 did not show any binding to all HB-EGF chimeric molecule-expressing cells (Fig. 5C). Thus, the epitope mapping experiment revealed that the region between amino acid residues 68 to 84 of the prodomain of HB-EGF constitutes the epitope of mAb 2-108 (Fig. 5A).

Our data showed that mAb 2-108 could recognize human HB-EGF but did not recognize mouse HB-EGF. In the region that constitutes the epitope of mAb 2-108 between amino acid residues 68 to 84, we identified six different amino acids between mouse HB-EGF and human HB-EGF. To further narrow down the epitope of mAb 2-108, we generated six-point mutants by replacing one of these specific six amino acids between residues 68 and 84 of human HB-EGF with the corresponding amino acid residue in mouse HB-EGF. The mutant proteins were expressed in HEK293 cells, and cell-binding ELISAs were performed (Fig. 5D). However, none of the single amino acid substitution mutants showed decreased binding by mAb 2-108, and thus, we could not identify a specific amino acid residue involved in the binding of mAb 2-108.

A previous study showed that Thr75 of human HB-EGF, which falls within the region between residues 68 to 84 identified above, is o-glycosylated.^(33)^ However, substitution of Thr75 to Ala (75T>A), which would abolish glycosylation at this residue, did not affect the reactivity to mAb 2-108 (Fig. 5D). We also digested the recombinant HB-EGF protein produced by HEK293 cells with an enzyme cocktail, including *o*-glycosidase, RNGaseF, neuraminidase, β1–4 galactosidase, and β-N-acetylglucosaminidase. However, the enzymatic treatment did not affect mAb 2-108 binding to HB-EGF by immunoblotting (data not shown). These results indicate that the glycosyl moiety of HB-EGF does not constitute the epitope for mAb 2-108 binding.

## Discussion

Despite longstanding efforts,^(23,27)^ no mAbs against HB-EGF have been obtained that are effective for IHC of paraffin-embedded specimens. In this study, we show that mAb 2-108 can be successfully used for IHC of paraffin-embedded specimens as well as other applications. This mAb possesses unique characteristics compared with other previously isolated anti-HB-EGF mAbs: (1) mAb 2-108 is capable of immunoblotting both under reducing and nonreducing conditions and (2) mAb 2-108 reacts with HB-EGF both in denatured and intact (unfixed) forms. This unique characteristic of mAb 2-108 seems to be due to the nature of the antigenic region of HB-EGF. Our results show that this mAb mainly recognizes the region between amino acid residues 68 and 84 of the prodomain. This region is structurally excluded from the EGF-like domain, which is constructed by sulfhydryl bonds,^(34)^ and thus, this region is allowed to be in a flexible form. Although previous studies have not isolated mAbs that recognize this region, here we obtained several hybridoma clones that produce mAbs that recognize this region of HB-EGF. In this study, the antigen used for immunization was produced in HEK293 cells, while previous studies used antigens produced in insect cells, and thus, this may contribute to our successful isolation of mAbs.

### Clinical value of mAb 2-108

HB-EGF is involved in the progression and malignancy of several cancers, including ovarian cancer, and is recognized as a therapeutic target for these cancers.^(11,12,26)^ Thus, mAbs clinically available for detection of HB-EGF expression would be an important tool in the therapeutic diagnosis of HB-EGF-related cancers. Clinical specimens obtained from surgical operations or biopsies are typically stored in paraffin-embedded blocks after fixation because of the ease and stability of this storage method. Thus, identifying an mAb that is applicable for IHC of paraffin-embedded sections has an immediate and direct value. Although the mAb 2-108 newly generated in this study can be successfully used for several applications, we notably confirmed that mAb 2-108 could successfully immunohistochemically detect HB-EGF in paraffin-embedded sections. The reactivity of this antibody was verified by various types of samples: cultured Vero-H cells, xenografted tumors of human ovarian cancer cells in nude mice, and cancerous tissues of ovarian cancer and other cancers. Normal adjacent tissues showed little staining with mAb 2-108, suggesting that the observed staining was not due to nonspecific binding of mAb 2-108 to tissues. Thus, our results demonstrate that mAb 2-108 is the first mAb against human HB-EGF available for IHC of paraffin-embedded sections and suggest that this antibody will be a powerful tool for the study and diagnosis of HB-EGF-related cancers and other diseases.

### Problem of N-terminal cleavage of HB-EGF by MT1-MMP

In this study, we showed that amino acid residues 68 to 84 of the prodomain of HB-EGF are necessary for the binding of mAb 2-108. Besides ectodomain shedding, HB-EGF is enzymatically processed in the prodomain.^(3)^ Our group previously reported that MT1-MMP cleaves off the N-terminal portion of HB-EGF at the site between 82Ala and 83Leu, and that this cleavage upregulates its mitogenicity in a heparin-independent manner.^(35)^ Indeed, a soluble HB-EGF fragment that corresponds to the MT1-MMP processed form was detected in malignant ascites obtained from patients with metastatic ovarian carcinoma.^(36)^ These findings indicate that cleavage of HB-EGF by MT1-MMP would result in a loss of the epitope recognized by mAb 2-108 and that mAb 2-108 would be unable to identify the processed short form of HB-EGF. Although information on the clinical importance of the processed short form is limited at this time, it is critical to note that mAb 2-108 does not recognize the short form of HB-EGF. It will be necessary to develop other novel mAbs that recognize the EGF-like domain of HB-EGF, which can be effective for IHC of paraffin-embedded tissues.
